# Supplementary material for: Effectiveness of COVID-19 Vaccines in the General Population of an Italian Region before and during the Omicron Wave
Source: Vaccines (Basel). 2022 Apr 22;10(5):662. doi: 10.3390/vaccines10050662 (PMC9146679; doi:10.3390/vaccines10050662)
Supplement: Supplementary file 1 [file vaccines-10-00662-s001.zip › vaccines-1652524-supplementary.pdf]

**Effectiveness of COVID-19 vaccines in the general population of an Italian Region before and during the Omicron wave - Online supplemental material**

**Table S1**. Schematic definition of the study groups, follow-up, and outcomes.

| | Unvaccinated | 1 Dose | 2 Doses | 3 Doses |
|---|---|---|---|---|
| **Inclusion criteria** | 1. Residency or domicile in the Abruzzo Region of Italy on 1 January 2020<br>2. No positive SARS-CoV-2 swab before the start of follow-up | | | |
| | 3. Did not receive COVID-19 vaccines. | 3. Received only one dose of BNT162b2, mRNA-1273 or ChAdOx1 nCoV-19 vaccines, from January 2, 2021 (date of the administration of the first vaccine dose), up to December 18, 2021 | 3. Received only one dose of JNJ-78436735 vaccine, or only two doses of BNT162b2, mRNA-1273 or ChAdOx1 nCoV-19 vaccines, from January 2, 2021 up to December 18, 2021 | 3. Received two doses of JNJ-78436735 vaccine, or three doses of BNT162b2, mRNA-1273 or ChAdOx1 nCoV-19 vaccines, from January 2, 2021 up to December 18, 2021 |
| **Follow-up** | For the comparison versus the group "1 dose", the follow-up started 14 days after the first administration of the first vaccine dose to the population (January 16, 2021)<br>For the comparison versus the group "2 doses", the follow-up started 14 days after the first administration of the second vaccine dose to the population (January 31, 2021)<br>For the comparison versus the group "3 doses", the follow-up started 14 days after the start of the mass administration of the third vaccine dose to the population (September 17, 2021) | The follow-up started 14 days after the single vaccine dose | The follow-up started 14 days after the second vaccine dose | The follow-up started 14 days after the third vaccine dose |
| | For all comparisons and groups, the follow-up ended the day of the outcome or on February 18, 2022, for those who did not experience the outcome | | | |
| **Outcomes** | 1. SARS-CoV-2 infection, detected through RT-PCR (Reverse transcription polymerase chain reaction, tested through nasopharyngeal swabs by the accredited laboratories of the Region)<br>2. Virologically-confirmed COVID-19 hospitalization (diagnosed by a specialist physician)<br>3. COVID-19-related death (inside or outside the hospital) | | | |

**Table S2.** Multivariable analysis Ψ predicting SARS-CoV-2 infection, COVID-19 hospitalization, and COVID-19-related death (complete results of the logistic models reported in Table 3 - Whole period, all subjects).

| | SARS-CoV-2 | COVID-19 hospitalization [A] | COVID-19-related death |
|---|---|---|---|
| | OR (95% CI) | OR (95% CI) | OR (95% CI) |
| *Vaccination* | | | |
| Unvaccinated | 1 (Ref. cat.) | 1 (Ref. cat.) | 1 (Ref. cat.) |
| Vaccinated (any dose) | 0.71 (0.70-0.72)* | 0.10 (0.09-0.11)* | 0.05 (0.04-0.06)* |
| Age class, years | | | |
| 60 or more | 1 (Ref. cat.) | 1 (Ref. cat.) | 1 (Ref. cat.) |
| 30-59 | 2.32 (2.28-2.36)* | 0.16 (0.14-0.19)* | 0.09 (0.07-0.11)* |
| 0-29 | 3.67 (3.61-3.74)* | 0.01 (0.01-0.02)* | NE |
| Male gender | 0.92 (0.91-0.93)* | 1.28 (1.17-1.40)* | 1.56 (1.37-1.77)* |
| Hypertension | 0.89 (0.87-0.91)* | 1.21 (1.08-1.36)** | 1.17 (1.00-1.35)** |
| Diabetes | 0.97 (0.94-1.00) | 1.37 (1.22-1.55)* | 1.36 (1.17-1.58)* |
| Major cardiovascular disease | 1.27 (1.23-1.30)* | 2.42 (2.15-2.71)* | 2.43 (2.09-2.82)* |
| Kidney disease | 0.92 (0.87-0.97)** | 1.23 (1.06-1.43)** | 1.00 (0.83-1.20) |
| COPD | 1.15 (1.11-1.20)* | 1.60 (1.40-1.84)* | 1.45 (1.22-1.72)* |
| Cancer | 0.85 (0.83-0.88)* | 1.11 (0.97-1.27) | 1.15 (0.98-1.36) |

OR = Odds Ratio; CI = Confidence Interval; Ref. cat. = Reference category. NE = Not estimable (0 cases in one or both of the groups under comparison). COPD: chronic obstructive pulmonary diseases. * $p<0.001$; ** $p<0.05$. Ψ Logistic regression models including 1,275,823 subjects (the total number of subjects included in the main comparison between 2 doses and no vaccine). [A] Virologically-confirmed COVID-19 disease, diagnosed by a specialist physician and requiring hospital admission.

**Table S3.** Multivariable analysis Ψ of the effectiveness of COVID-19 vaccines during pre-Omicron waves [A].

| | SARS-CoV-2 | COVID-19 hospitalization [B] | COVID-19-related death |
|---|---|---|---|
| | OR (95% CI) | OR (95% CI) | OR (95% CI) |
| **Health status** | | | |
| *Vaccine doses* | | | |
| All subjects | | | |
| Unvaccinated | 1 (Ref. cat.) | 1 (Ref. cat.) | 1 (Ref. cat.) |
| 1 dose | 0.57 (0.53-0.61)* | 0.46 (0.34-0.62)* | 0.46 (0.32-0.64)* |
| 2 doses | 0.25 (0.24-0.25)* | 0.04 (0.03-0.05)* | 0.04 (0.03-0.05)* |
| 3 doses | 0.12 (0.10-0.13)* | 0.03 (0.01-0.09)* | 0.02 (0.00-0.12)* |
| Infected subjects only | | | |
| Unvaccinated | -- | 1 (Ref. cat.) | 1 (Ref. cat.) |
| 1 dose | -- | 0.40 (0.28-0.57)* | 0.28 (0.19-0.43)* |
| 2 doses | -- | 0.07 (0.06-0.09)* | 0.05 (0.04-0.07)* |
| 3 doses | -- | 0.15 (0.05-0.44)* | 0.06 (0.01-0.43)* |
| **Vaccine type** (2 doses vs. none) | | | |
| Unvaccinated | 1 (Ref. cat.) | 1 (Ref. cat.) | 1 (Ref. cat.) |
| BNT162b2 | 0.25 (0.24-0.25)* | 0.06 (0.05-0.08)* | 0.05 (0.04-0.07)* |
| mRNA-1273 | 0.14 (0.13-0.15)* | 0.03 (0.01-0.06)* | 0.02 (0.01-0.06)* |
| ChAdOx1 nCoV-19 | 0.42 (0.40-0.44)** | 0.01 (0.00-0.02)* | 0.01 (0.00-0.02)* |
| JNJ-78436735 | 0.57 (0.51-0.64)* | 0.03 (0.00-0.18)* | 0.00 (NE) |
| Mixed [C] | 0.24 (0.21-0.28)* | 0.03 (0.00-0.23)* | 0.04 (0.01-0.30)* |
| **Age-class**, years (2 doses vs. none) | | | |
| 60 or more | 0.33 (0.31-0.35)* | 0.05 (0.04-0.06)* | 0.04 (0.03-0.05)* |
| 30-59 | 0.29 (0.27-0.30)* | 0.02 (0.01-0.04)* | 0.01 (0.00-0.03)* |
| 0-29 | 0.16 (0.15-0.17)* | 0.08 (0.02-0.39)* | NE |

OR = Odds Ratio; CI = Confidence Interval; Ref. cat. = Reference category. NE = Not estimable (0 cases in one or both of the groups under comparison). * $p<0.001$. Ψ Logistic regression models adjusted for age, gender, hypertension, diabetes, major cardiovascular diseases, chronic obstructive pulmonary diseases, kidney diseases, and cancer. [A] Including only the outcomes that occurred from the start of follow-up to December 26, 2021, when the proportion of Omicron variant in the available positive swabs was lower than 50%. [B] Virologically-confirmed COVID-19 disease, diagnosed by a specialist physician and requiring hospital admission. [C] Subjects who received two or three different vaccines.
